# Validation of a PCR test to predict the presence of flavor volatiles mesifurane and γ-decalactone in fruits of cultivated strawberry (*Fragaria* × *ananassa*)

**DOI:** 10.1007/s11032-017-0732-7

**Published:** 2017-10-02

**Authors:** Eduardo Cruz-Rus, Rafael Sesmero, José A. Ángel-Pérez, José F. Sánchez-Sevilla, Detlef Ulrich, Iraida Amaya

**Affiliations:** 1 Instituto Andaluz de Investigación y Formación Agraria y Pesquera (IFAPA) Centro de Churriana, Cortijo de la Cruz, 29140 Málaga, Spain; 2 Present Address: Departamento de Biología Vegetal, Universidad de Málaga, 29071 Málaga, Spain; 3 Institute for Ecological Chemistry, Plant Analysis and Stored Product Protection, Julius Kühn-Institute (JKI), Federal Research Centre for Cultivated Plants, Erwin-Baur-Str. 26, 06484 Quedlinburg, Germany

**Keywords:** Molecular marker, Genetic screening, *Fragaria*, Flavor, Aroma, Marker-assisted selection, Breeding

## Abstract

Flavor improvement is currently one of the most important goals for strawberry breeders. At the same time, it is one of the most complex traits to improve, involving the balanced combination of several desired characteristics such as high sweetness, moderate acidity, and the appropriate combination of aroma compounds that are beginning to be delineated in consumer tests. DNA-informed breeding will expedite the selection of complex traits, such as flavor, over traditional phenotypic evaluation, particularly when markers linked to several traits of interests are combined during the breeding process. Natural variation in mesifurane and γ-decalactone, two key volatile compounds providing sweet Sherry and fresh peach-like notes to strawberry fruits, is controlled by the *FaOMT* and *FaFAD1* genes, respectively. In this study, we have optimized a simple PCR test for combined analysis of these genes and determined a prediction accuracy above 91% using a set of 71 diverse strawberry accessions. This high accuracy in predicting the presence of these important volatiles combined with the simplicity of the analytical methodology makes this DNA test an efficient tool for its implementation in current strawberry-breeding programs for the selection of new strawberry cultivars with superior flavor.

**Electronic supplementary material:**

The online version of this article (10.1007/s11032-017-0732-7) contains supplementary material, which is available to authorized users.

## Introduction

Strawberry is the fruit with the highest production among berries with a global production of over eight million tons (FAOSTAT 2017). The cultivated strawberry (*Fragaria* × *ananassa*) is a relatively new species originated by the accidental hybridization of South American *Fragaria chiloensis* and North American *Fragaria virginiana* in a European garden about 250 years ago (Hancock 1999). Systematic breeding of strawberry began shortly afterward and has become an area of substantial economical importance. However, strawberry genetics and breeding is complex due to its octoploid genome (2n = 8× = 56), which is thought to have originated through interspecific hybridization (allopolyploidy) involving up to four species (Rousseau-Gueutin et al. 2009; Tennessen et al. 2014). Recent studies indicate that one of the four subgenomes originates from the diploid donor *F*. *vesca*, one from the diploid *F*. *iinumae*, and the remaining two from an unknown ancestor close to *F*. *iinumae* (Tennessen et al. 2014). These and other studies have shown that the genome of *F*. *vesca* (Shulaev et al. 2011) can be used as a reference for genomic studies but also that the genome of octoploid *Fragaria* species has suffered typical genomic changes after polyploidization such as gene loss and genetic rearrangements between the four subgenomes (Rousseau-Gueutin et al. 2008; Sargent et al. 2012; Tennessen et al. 2014; Sánchez-Sevilla et al. 2015). Due to the possibility of up to eight alleles from four homoeologous loci contributing to the variation in each particular gene, improving agronomical and fruit quality traits can be extremely challenging in this species (Lecerteau-Köhler et al. 2012).

Traditionally, strawberries have been highly appreciated for their flavor, which results from a combination of sugars, acids, and volatile organic compounds (VOCs). However, breeding efforts during the last two centuries have been very intensive in industry-demanded targets such as fruit size and shape, yield, pest resistance, fruit firmness, and postharvest life, improvements that have sometimes come at the expense of sensory qualities (Folta and Klee 2016; Ulrich and Olbricht 2016). This tendency is changing; consumer preferences are now taken into account, and the evaluation of total sugar content using soluble solid content (SSC) and acidity in fruits are included at different time points during breeding programs. However, volatile evaluation is expensive, not amenable to high-throughput assays, and as a result, aroma is more difficult to breed for and beyond the scope of the majority of current breeding programs.

Despite the interest in fruit flavor improvement, markers linked to this complex trait are difficult to develop due to the complexity of the different primary and secondary biochemical pathways involved in the biosynthesis of these compounds (Klee 2010). Moreover, volatile patterns in a particular cultivar are also regulated by developmental and environmental cues (Jouquand et al. 2008; Olbricht et al. 2011; Schwieterman et al. 2014). More than 360 VOCs have been identified in strawberry, and the definition of the key chemical constituents of strawberry aroma (character impact compounds) varies among different studies. Three different approaches have been used to estimate character impact compounds, i.e., (1) the relation of quantity and odor thresholds for human perception (aroma value concept), (2) gas chromatography-olfactometry, and (3) quantity and correlation with sensory attributes by consumer test panels (Larsen and Poll 1992; Larsen et al. 1992; Schieberle and Hofmann 1997; Ulrich et al. 1997; Prat et al. 2014; Schwieterman et al. 2014; Ulrich and Olbricht 2016). In general, common VOCs across the different studies contributing to the fruity flavor of strawberry are esters such as methyl and ethyl butanoates, and methyl and ethyl hexanoates. Terpene linalool has been linked to flowery and sweet aroma and two furanones: furaneol (2,5-dimethyl-4-hydroxy-3(2*H*)-furanone; DMHF) and mesifurane (2,5-dimethyl-4-methoxy-3(2*H*)-furanone; DMMF), to fruity and caramel aromas. Other key compounds are γ-decalactone and γ-dodecalactone, contributing to fresh peachy aroma and which have been associated with increased perception of sweetness in the fruit (Schwieterman et al. 2014; Ulrich and Olbricht 2016). This is particularly important since total sugar content in the fruit has been negatively correlated with yield parameters (Zorrilla-Fontanesi et al. 2011; Whitaker et al. 2012). Therefore, an alternative solution to increase strawberry sweetness perception without affecting yield is breeding for VOCs acting as sweet enhancers such as γ-decalactone (Schwieterman et al. 2014).

A comprehensive study in the 232 ***×*** 1392 mapping population identified QTLs controlling 48 different VOCs during different seasons (Zorrilla-Fontanesi et al. 2012). This study highlighted a high stability of about 50% of the QTLs when grown in the same cultivation system during three consecutive years. Natural variation in the content of two key VOCs, mesifurane and γ-decalactone, is controlled by major genes, enabling their identification (Zorrilla-Fontanesi et al. 2012). A combination of metabolomics and expression studies in the progeny lines of this mapping population resulted in the identification of *FaOMT* as the gene controlling natural variation in mesifurane content in strawberry (Zorrilla-Fontanesi et al. 2012). Sequence analysis of different *FaOMT* promoter alleles from progeny lines with contrasting phenotypes identified a 30-bp indel in the proximal region containing three potential cis-regulatory elements (E-box/RRE motif, MYBL motif, and an ABRE/ACGT motif) that were specific to the functional allele (Zorrilla-Fontanesi et al. 2012). A marker, FaOMT-SI/NO, flanking this polymorphism was developed, and analysis in the parental and the 95 F1 progeny lines resulted in 100% co-segregation of mesifurane presence and the amplification of an allele of 248 bp. In contrast, the inactive allele, containing the 30 bp deletion, was amplified by FaOMT-SI/NO marker as a band of 217 bp. Since cultivated strawberry is an allo-octoploid, a number of larger *FaOMT* alleles not related with the phenotype were also amplified, representing most probably homoeologs from the other three subgenomes. The parental lines 232 and 1392 were both heterozygous for the active and the inactive alleles, and these two marker alleles fitted a 1:2:1 segregation in the F1 population. Therefore, this marker could represent a useful tool for the selection of strawberry cultivars with high concentration, or alternatively without mesifurane, in the fruit.

Using the same mapping population and a genome-wide transcriptome analysis by RNA-seq of two bulked pools of progeny lines contrasting in the content of γ-decalactone, *FaFAD1* was identified as a key gene controlling this important VOC (Sánchez-Sevilla et al. 2014). Simultaneously, another group using complementary approaches in a different segregating population identified the same gene required to synthesize γ-decalactone, which provides “peachy” notes in strawberry (Chambers et al. 2014). Both studies provided evidences that *FaFAD1* was essential, as a number of different lines not able to accumulate γ-decalactone in their fruits presented either a complete deletion of this gene or a radically different sequence, as several primer pairs were not able to detect the gene by PCR (Chambers et al. 2014; Sánchez-Sevilla et al. 2014).

A DNA test to predict the presence/absence of important VOCs in fruits could improve selection efficiency during breeding programs and facilitate the selection of new tastier cultivars. A simple DNA test that can reliably predict the phenotype across diverse germplasm and parental lines is a useful tool for its routine application in strawberry breeding. In order to reduce the cost and labor of volatile phenotyping, we selected a core collection of cultivars representing the genetic diversity of the species based on analysis of diversity and genetic structure. Here, we report the development of a single diagnostic test for both VOCs mesifurane and γ-decalactone and validate its predictive capacity in a diverse collection of strawberry cultivars.

## Material and methods

### Plant material, DNA isolation, and volatile extraction

Strawberry accessions used in this study were collected at the IFAPA strawberry germplasm collection (ESP138) located at Centro IFAPA de Churriana, Málaga, Spain (Table 1). A total of 71 accessions were selected to represent both a broad view of the cultivated strawberry varieties and a significant representation of the cultivars adapted to Mediterranean/Californian climates, which are commonly used founders in current breeding programs and the most actively commercialized cultivars worldwide (Faedi et al. 2002; López-Aranda et al. 2011). About half of the 71 accessions have been selected based on previous studies of genetic diversity in strawberry (Gil-Ariza et al. 2009; Horvath et al. 2011; Sánchez-Sevilla et al. 2015). Likewise, 61 of the 71 accessions were selected based on their distribution across a wide range of different clusters of a Neighbor-Joining tree with a total of 176 strawberry accessions genotyped using 21,971 SNPs (unpublished results) from the IStraw90 Axiom® array (Bassil and Davis et al. 2015). A Neighbor-Joining clustering of the selected 61 strawberry accessions is shown in Online Resource 1. Three plants per accession were grown under greenhouse conditions and young leaf samples and ripe fruits were harvested for DNA isolation and volatile extractions, respectively. For fruit volatile extractions, ripe fruits were harvested a number of times during the season to ensure at least 20 ripe fruits from each genotype. Leaf and fruit samples were immediately frozen in liquid nitrogen and stored at − 80 °C until further analyses.

**Table 1:** List of strawberry cultivars and other *Fragaria* species used, mesifurane (DMMF) and γ-decalactone (γ-DEC) content (raw data in peak areas) and *FaOMT* and *FaFAD1* genotypes. Non-matching results are in italics. ACCID, accession ID at the IFAPA Germplasm bank; –, no data; yes, indicates the presence of volatile when measured in the cited study using different units.

Name	ACCID	Date	Country	DMMF	FaOMT	γ-DEC	*FaFAD1*	Reference^b^
232	–	1996	Spain	Yes	248/217 bp	0.00	No	[1]
1392	–	2002	Spain	Yes	248/217 bp	Yes	140 bp	[1]
93-23	–	2006	Spain	0.00	217 bp	Yes	140 bp	[1]
93-34	–	2006	Spain	0.00	217 bp	0.00	No	[1]
93-10	–	2006	Spain	Yes	248 bp	0.00	No	[1]
93-47	–	2006	Spain	Yes	248 bp	Yes	140 bp	[1]
Aguedilla	519	2003	Spain	0.00	217 bp	2.10	No	[2]
Amiga	480	2006	Spain	13.86	248/217 bp	0.00	No	[3]
Aromas	300	2010	USA	*0.00*	*248/217 bp*	38.65	140 bp	[3]
Proprietary cultivar 1^a^	288	1998	USA	5.69	248/217 bp	0.00	No	[3]
Camarosa	72	1992	USA	30.66	248/217 bp	0.00	No	[3]
Camino real	677	2003	USA	24.81	248/217 bp	70.78	140 bp	[3]
Candonga	715	2003	Spain	*0.00*	*248/217 bp*	51.70	140 bp	[3]
Proprietary cultivar 2^a^	292	1997	USA	144.93	248 bp	0.00	No	[3]
Chandler	84	1983	USA	12.73	248/217 bp	189.09	140 bp	[3]
Cifrance	664	1996	France	30.97	248/217 bp	281.51	140 bp	[3]
Cijosee (or Cireane)	301	1997	France	33.23	248/217 bp	89.14	140 bp	[3]
Commitment	701	2004	USA	7.28	248/217 bp	0.00	No	[3]
Coral	778	1993	Romania	41.18	248 bp	183.75	140 bp	[3]
Deutsch Evern	304	1902	Germany	0.00	217 bp	0.00	No	[3]
Diamante	302	1995	USA	10.58	248/217 bp	112.95	140 bp	[3]
Douglas	130	1979	USA	33.50	248/217 bp	0.00	No	[3]
Proprietary cultivar 3^a^	956	2010	USA	140.12	248 bp	0.00	No	[3]
Proprietary cultivar 4^a^	955	2010	USA	641.34	248 bp	*11.40*	*No*	[3]
Elvira	126	1967	NDL	*0.00*	*248 bp*	0.00	No	[4]
Endurance	699	2004	USA	123.27	248 bp	0.00	No	[3]
Everest	315	1971	UK	30.31	248/217 bp	0.00	No	[3]
Festival	732	2000	USA	7.13	248/217 bp	0.00	No	[3]
Fuentepina	879	2009	Spain	38.37	248/217 bp	310.39	140 bp	[3]
Galante	678	2004	USA	13.72	248/217 bp	*0.00*	*140 bp*	[3]
Galexia	733	2004	USA	8.46	248/217 bp	26.08	140 bp	[3]
Gento	131	1967	Germany	19.27	248/217 bp	*13.71*	*No*	[3]
Gigantella	308	–	NDL	20.30	248/217 bp	0.00	No	[3]
Honor	700	2004	USA	101.04	248 bp	0.00	No	[3]
Hood	832	1965	USA	0.00	217 bp	0.00	No	[3]
Jucunda	843	1854	UK	172.70	248 bp	0.00	No	[3]
Proprietary cultivar 5^a^	854	2005	USA	131.08	248 bp	0.00	No	[3]
Macarena	777	2004	Spain	0.00	217 bp	0.00	No	[3]
Mara des bois	213	1992	France	76.72	248 bp	0.00	No	[3]
Medina	433	2002	Spain	18.74	248/217 bp	0.00	No	[3]
Mieze Schindler	307	1933	Germany	0.00	217 bp	0.00	No	[5]
Milsei	77	1990	Spain	4.88	248/217 bp	78.51	140 bp	[3]
Naiad	697	2000	Italy	35.08	248/217 bp	9.94	140 bp	[3]
Oso Grande	104	1987	USA	162.60	248/217 bp	*9.14*	*No*	[3]
Palomar	856	2007	USA	12.61	248/217 bp	72.98	140 bp	[3]
Pandora	39	1988	UK	0.00	217 bp	Yes	140 bp	[4]
Parker	110	1984	USA	82.10	248/217 bp	3.14	No	[3]
Pedrone	809	2004	Spain	*0.00*	*248/217 bp*	57.80	140 bp	[3]
Plarionfre (or Chiflon)	674	1999	Spain	38.77	248/217 bp	5.59	No	[3]
Premial	316	1989	Romania	0.00	217 bp	70.13	140 bp	[3]
Reusraths aller Krüester	844	1949	Germany	37.00	248 bp	0.00	No	[3]
Roxana	706	2000	Italy	18.29	248/217 bp	0.00	No	[3]
Rubygem	839	2004	Australia	64.20	248/217 bp	218.31	140 bp	[3]
Ruby	673	1998	USA	75.77	248/217 bp	0.00	No	[3]
Sabrina	965	2009	Spain	52.32	248/217 bp	555.89	140 bp	[3]
Santaclara	884	2009	Spain	33.50	248/217 bp	699.80	140 bp	[2]
Splendor	862	2005	USA	11.50	248/217 bp	0.00	No	[3]
Tioga	112	1964	USA	38.86	248/217 bp	2.78	No	[3]
Toyonoka	245	1975	Japan	0.00	217 bp	0.00	No	[3]
Ventana	676	1997	USA	0.00	217 bp	29.22	140 bp	[3]
Ville de Paris	294	1929	France	58.30	248/217 bp	0.00	No	[3]
Virtude	866	2005	USA	18.67	248/217 bp	0.00	No	[3]
Viva Patricia	953	2009	UK	132.80	248 bp	339.13	140 bp	[3]
Winter Dawn	838	2009	USA	6.05	248/217 bp	0.00	No	[3]
Proprietary cultivar 6^a^	290	1997	USA	90.54	248 bp	12.35	140 bp	[3]
CS 9/2 (*F*. *chiloensis* × ‘Ventana’)	–	2004	Spain	6.35	248/217 bp	*10.17*	*No*	[3]
CS 13/2 (‘Camarosa’ × *F*. *chiloensis*)	–	2005	Spain	0.00	217 bp	0.00	No	[3]
*F*. *virginiana* UC-11 (Corvalis PI 551495)	180	1974	USA	*735.49*	*None*	12.24	140 bp	[3]
*F. moschata* ‘Capron Royale’	591	–	France	*348.61*	*None*	23.36	140 bp	[3]
*F*. *vesca* Blanca	596	–	–	*6.95*	*None*	*0.00*	*140 bp*	[3]
*F*. *vesca* ‘Reine des vallées’	660	–	France	0.00	None	28.38	140 bp	[3]

^a^Driscoll’s proprietary material

^b^Phenotype for volatiles obtained in: [1], Zorrilla-Fontanesi et al. 2012; [2], unpublished study; [3], this study; [4], Larsen et al. 1992; [5], Ulrich et al. 1997

Total genomic DNA from strawberry accessions was isolated from 130 mg of young unexpanded leaf samples, ground in liquid nitrogen, using a modified CTAB method based on that of Doyle and Doyle (1990). DNA concentration was quantified at 260 nm using a NanoDrop spectrophotometer (ND-1000 V3.5, NanoDrop Technologies, Inc.), and its quality was checked by two absorbance ratios, 260/230 and 260/280 nm, and by 0.7% (*w*/*v*) agarose gel electrophoresis.

Sample for semi-quantitation of VOCs were prepared from ripe strawberry fruits stored at − 80 °C after harvest. To prepare an enzyme-inhibited strawberry juice, a minimum of 200 g berries from each genotype was used. One mass part of fruits was homogenized in one volume part of a solution of 18.6% (*m*/*v*) NaCl using a Waring blender for 2 min. The homogenate was centrifuged 4000 rpm for 20 min at 4 °C. One hundred milliliter of the supernatant was mixed with 10 μL internal standard (0.1% (*v*/*v*) 2,6-dimethyl-5-hepten-2-ol dissolved in ethanol). For each sample, three 20-mL-headspace vials containing 3 g NaCl each for saturation were filled with 10 mL of the supernatant, sealed with magnetic crimp caps including septum, and stored at 4 °C.

### PCR amplification and gel electrophoresis

Primers used for allele discrimination in genes *FaOMT* and *FaFAD1* were developed previously (Zorrilla-Fontanesi et al. 2012; Sánchez-Sevilla et al. 2014). The only difference is that for this work, the forward primer FaOMT-SI/NO-F for gene *FaOMT* was extended with two additional nucleotides (Online Resource 2). PCRs for individual gene assays were performed in a final reaction volume of 15 μL containing 2 μL (~10 ng) of template DNA, 3 μL ×5 Taq PCR buffer (GoTaq® buffer, Promega Corp., Madison, WI), 200 μm deoxyribonucleotide triphosphates (dNTP), 0.2 μm of each forward and reverse primer, and 0.5 U Taq polymerase (GoTaq®, Promega Corp., Madison, WI). As positive control of PCR for the dominant marker qFaFAD1, primers q2-FaM6PI-F (5′-CGAGTTTGAGGTCGATCGGT-3′) and FaM6PI-3UTR (5′-ACAGCTTGTTTGCATCTTCCAA-3′) were included in the reaction at a concentration of 0.2 μm each. This primer pair produced an amplicon of 300 bp in all tested accessions allowing differentiation between negative *FaFAD1* amplification and total PCR failure. For combined *FaOMT/FaFAD1* assays, alleles of *FaOMT* served as positive PCR controls for the dominant *FaFAD1* marker, simplifying the assay to a combination of only two primer pairs. PCRs were performed in a final reaction volume of 20 μL containing the same amount of template DNA as individual assays, 4 μL ×5 Taq PCR buffer, 200 μm deoxyribonucleotide triphosphates (dNTP), 0.1 μm each of the four primers, and 0.5 U Taq polymerase. PCR conditions followed a touchdown protocol as follows: 95 °C for 3 min followed by 10 cycles of 95 °C for 30 s, 60 °C (− 0.5 °C/cycle) for 30 s, and 72 °C for 45 s, then 25 cycles of 95 °C for 30 s, 55 °C for 30 s, and 72 °C for 45 s, followed by a final extension at 72 °C for 5 min.

Gel electrophoresis of PCR samples was carried out using 2% agarose gels, containing 6 μL ×20,000 RedSafe staining solution (Ecogen, Spain) per 100 mL gel, in TBE buffer under standard electrophoresis conditions.

### Semi-quantitation using HS-SPME-GC-FID

Volatiles were sampled by HS-SPME using a 100-mm polydimethylsiloxane fiber (Supelco, Bellefonte, PA, USA). Initially, vials were equilibrated at 35 °C for 10 min in the shaking operation mode (300 rpm). Then, the volatiles were extracted by exposing the fiber to the vial headspace for 15 min at 35 °C under continuous agitation. Thermal desorption was programmed for 2 min in the injector (splitless mode) at 250 °C, and followed by additional thermal cleaning for 3 min at 250 °C (split ratio 1:10). Incubation of the vials, extraction, and desorption of the volatiles were performed automatically by a MPS2 autosampler from Gerstel (Mühlheim an der Ruhr, Germany).

Chromatography was carried out using an Agilent Technologies 6890 gas chromatograph (Agilent Technologies Deutschland GmbH, Böblingen, Germany) equipped with a flame ionization detector (FID). Compounds were separated on a polar column HP INNOWax, 0.25 mm ID × 30 m length × 0.5 μm film thickness with hydrogen as carrier gas at a constant flow of 1.1 mL/min. The FID temperature was set at 250 °C. Oven temperature conditions were 40 °C for 3 min, a ramp from 40 to 200 °C at 3 K/min and 15 min at 200 °C. Samples were run in triplicate.

For substance identification, parallel runs of selected samples were performed using a similar GC instrument equipped with an Agilent 5973 MSD in the electron impact ionization mode (70 eV) under the same conditions and with helium as carrier gas. Chromatograms and spectra were recorded and processed using the Enhanced ChemStation software (Agilent Technologies). Compounds were identified by comparison of their mass spectrum and retention indices to those in the Wiley and NIST05 library, and also by co-elution and comparison of mass spectrum and retention time to those of pure standards (SIGMA-Aldrich; for all VOCs except bisabolol oxide). Total ion chromatograms were integrated using the Agilent Chemstation to obtain peak areas.

### Statistical analysis

Principal component analysis (PCA) and construction of the box plots were performed with the software STATISTICA 7.1 by Statsoft using raw data (peak areas in counts). For the assembly of the heat map, the software Multi Experiment Viewer 4.8.1 (http://www.tm4.org/) was used with the option of a hierarchical cluster analysis. Test prediction performance for FaOMT-SI/NO and qFaFAD1 markers was analyzed in relation with the presence/absence of mesifurane and γ-decalactone, respectively. Estimates of accuracy, positive predictive value (PPV), negative predictive value (NPV), sensitivity (or true positive rate), specificity (or true negative rate), and adjusted diagnostic odds ratio (ADOR) were calculated according to Salinas and Zurn et al. (2017). These metrics were calculated for 67 and 71 accessions for FaOMT-SI/NO and qFaFAD1 markers, respectively, and are used as quantitative indicators of the test’s ability for accurate diagnosis (Aliu and Chung 2012; Glas et al. 2003). The indicators PPV, NPV, sensitivity, and specificity represent only part of the discriminatory evidence, as high sensitivity may be accompanied by low specificity. In contrast, the ADOR statistic combines the strengths of sensitivity and specificity with the advantage of accuracy as a single indicator (Glas et al., 2003). ADOR accounts for the strength of the association between test result and phenotype and is defined as [(*ADJ sensitivity* × *ADJ specificity*)/(*1 − ADJ sensitivity*) × (*1 − ADJ specificity*))], where adjusted sensitivity and specificity values resulted from adding 0.5 to every cell of the contingency table (Salinas and Zurn et al. 2017; Glas et al., 2003). ADOR values range from 0 to infinity, with higher values indicating better discriminatory test performance.

### FaOMT promoter isolation and analysis

For characterization of the *FaOMT* promoter fragment from selected strawberry accessions, PCR and separation in agarose electrophoresis were performed as previously described for FaOMT-SI/NO marker test. Selected bands of about 500 bp from *F*. *virginiana* UC-11 and *F. moschata* ‘Capron Royale’, and 248 bp from *F*. × *ananassa* cv. ‘Aromas’, ‘Candonga’, ‘Elvira’, and ‘Pedrone’ were isolated and purified from the agarose gel using the FavorPrep GEL/PCR purification kit (Favorgen Biotech Corp.) and cloned into the pGEM-T Easy vector (Promega). Five independent clones were sequenced for each of the three accessions and assembled into individual contigs using the SeqMan tool (DNAStar). Sequence analyses and comparisons were carried out using the Lasergen software (DNAStar) and the tool Clustal W2 from EBI-EMBL.

## Results

A total of 71 strawberry accessions including 65 *F*. × *ananassa* cultivars, 2 *F*. *vesca* samples, 1 *F*. *moschata*, 1 *F*. *virginiana*, and 2 hybrids between *F*. × *ananassa* and *F*. *chiloensis* were used for marker validation (Table 1). Among the collection of 71 selected accessions, the 65 strawberry cultivars included samples from different breeding programs worldwide, including cultivars adapted to cultivation in the United States of America (USA), Germany, France, Spain, Japan, and Australia. This comprehensive panel included historically important cultivars and progenitors from different origins and also cultivars whose fruits have been described as highly aromatic, such as ‘Mara des bois’ and ‘Mieze Schindler’ (Ulrich and Olbricht 2016). The accessions from the related *Fragaria* species were selected based on the high aroma of their fruits, similar to the two hybrids with *F*. *chiloensis* (CS9/2 and CS13/2), which have been used as initial breeding lines within the IFAPA program. The wide diversity of this set of accessions is illustrated as a Neighbor-Joining clustering in Online Resource 1 using 61 out of the 71 accessions (see “Material and methods”). In this analysis, the selected 61 strawberry accessions were distributed into six clusters with different levels of admixture to the ancestry groups of the complete set of 176 accessions (Online Resource 1).

### Phenotypic variation in fruit volatile organic compounds

**Fig. 1:**
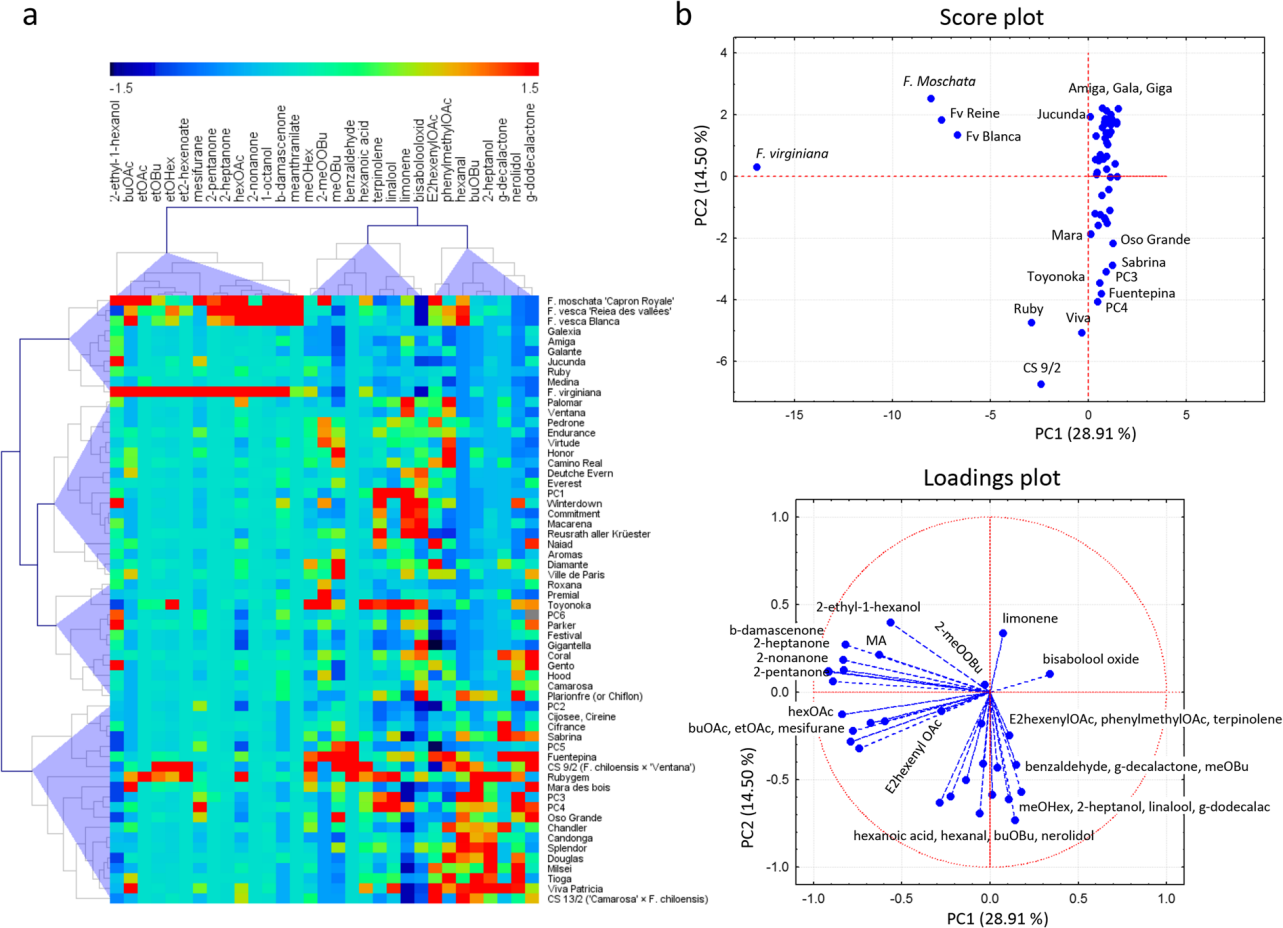
**a** Heat map grouped by a hierarchical cluster analysis (HCL) of the VOC composition of 60 samples and derived hierarchy of both cultivar and metabolite relatedness is displayed vertically and horizontally, respectively. **b** Score plot and loading plot of a principal component analysis (PCA)

Fruit volatiles from the strawberry panel were semi-quantified using headspace solid phase microextration (HS-SPME) sampling coupled to gas chromatography-mass spectrometry (GC-MS), although the data from the 232 × 1392 segregating population, ‘Elvira’, ‘Pandora’, and ‘Mieze Schindler’, were previously obtained and reported and data from ‘Aguedilla’ and ‘Santaclara’ was obtained from a different ongoing study (Table 1). The quantitative range of the VOC variation in the strawberry collection was remarkable. In Online Resource 3, the range of metabolite variation is depicted as box plots. Out of the 31 VOCs, 25 (80.6%), including mesifurane and γ-decalactone, show qualitative effects of substance contents, that is in one or more accessions zero values for the distinct compound were found (Online Resource 4). The most abundant compound was ethyl hexanoate. Regarding mesifurane and γ-decalactone, 47 accessions (78.3%) and 30 accessions (50%), respectively, contained these compounds while it was not significantly produced in the remaining samples (below the detection threshold of 6.0). Metabolite variation is visualized by a heat map and a PCA in Fig. 1. In the heat map (Fig. 1a), accessions as well as VOCs are clustered using a hierarchical cluster analysis. As shown in other reports, the accessions from *F*. *vesca*, *F*. *moschata*, and *F*. *virginiana* were characterized for a different set of VOCs compared to cultivars of *F*. × *ananassa* (Ulrich et al. 2007; Ulrich and Olbricht 2013; Ulrich and Olbricht 2014; Negri et al. 2015). For example, the content of ketones and octanol was higher while the content of nerolidol and γ-dodecalactone was lower in these wild species compared to *F*. × *ananassa* cultivars. The ester methyl anthranilate, responsible for the intense sweet and flowery notes of wild strawberries, was only detected in low levels of 5.51 and 5.01 (relative concentration units in peak area) in two *F*. × *ananassa* accessions: ‘Mara des bois’ and the hybrid with *F*. *chiloensis* CS9/2, while showing high accumulation in wild species, ranging from 47.72 in *F*. *virginiana* to 412 in *F*. *moschata* (Fig. 1a; Online Resource 4; Ulrich et al. 2007; Ulrich and Olbricht 2013; Negri et al. 2015). Thus, the hierarchical cluster analysis classified the 60 assayed accessions into four clusters, with the four accessions of the three wild species integrated in the top cluster, whereas the two *F*. *chiloensis* hybrids grouped in the bottom cluster. As a result of these differences in volatile composition, PCA distributed the majority of cultivars in a stretched cluster while wild species and one of the two hybrids were clearly separated (Fig. 1b).

When using available data for the complete set of 71 accessions, approximately three quarters (76%) of the 71 accessions (54) contained mesifurane in their fruits while 17 samples (24%) did not produce detectable amounts of this volatile. Regarding γ-decalactone, 32 accessions (45%) presented the volatile in different quantities while it was not significantly produced (below the detection threshold of 6.0) in 39 (55%) strawberry accessions (Table 1).

### Genotyping using FaOMT-SI/NO and qFaFAD1 markers

**Fig. 2:**
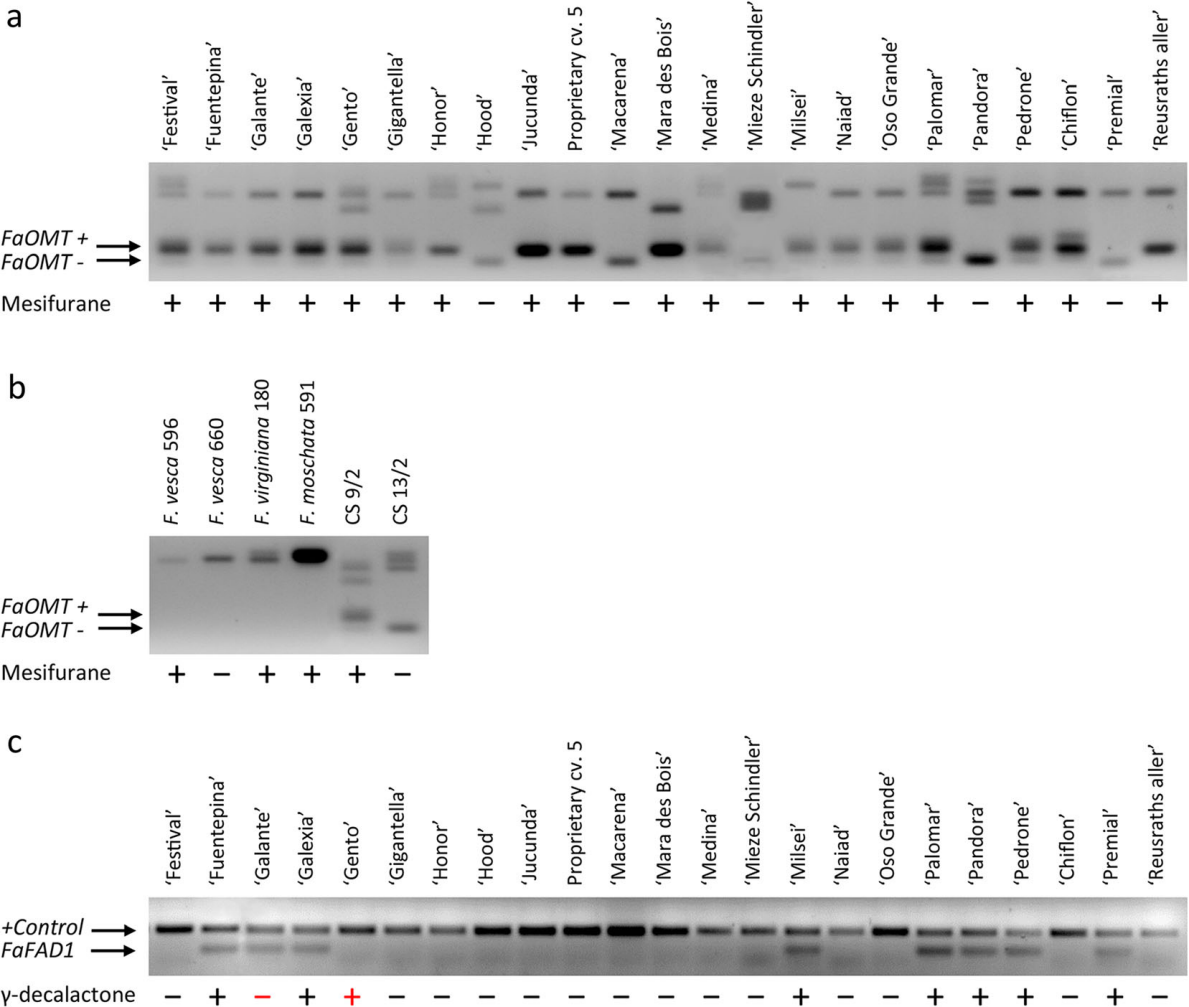
Examples of PCR bands amplified using FaOMT-SI/NO (**a**, **b**) and qFaFAD1 (**c**) markers in representative samples separated in 2% agarose gels. Symbols + and − indicates presence and absence of mesifurane or g-decalactone in their fruits, respectively

For genotyping, the 71 strawberry accessions were first PCR tested for amplification of the FaOMT-SI/NO and qFaFAD1 specific markers independently (Fig. 2). The FaOMT-SI/NO marker allows the discrimination of active and inactive alleles of the *FaOMT* gene based on their amplification size, 248 and 217 bp, respectively (Zorrilla-Fontanesi et al. 2012). However, this marker did not amplify the predictive 217/248 bp bands in the four samples representing the three wild species (Fig. 2b). Instead, larger bands corresponding to other alleles also present in the *F*. × *ananassa* accessions were amplified in these species, as previously reported for the 232 × 1392 population (Zorrilla-Fontanesi et al. 2012). Among the remaining 67 samples, including the two hybrids between *F*. × *ananassa* and *F*. *chiloensis*, 15 (22.4%) were homozygous for the active 248 bp allele, 40 (59.7%) were heterozygous (presenting both 248 and 217 bp bands), and 12 (17.9%) were homozygous for the inactive (217 bp) allele (Table 1). In heterozygous samples, the inactive 217 bp allele is amplified less efficiently than the 248 bp allele, resulting in an extremely faint band of 217 bp and difficult discrimination between 248/248 and 248/217 genotypes. However, heterozygous samples present an additional and also faint heteroduplex band that migrates above the 248 bp allele, which allows an efficient determination of the allelic composition of these lines (Fig. 2a).

The qFaFAD1 marker was designed to amplify a 140-bp fragment within the second exon of the *FaFAD1* gene. Previous analyses in lines of the 232 × 1392 population, cultivars such as ‘Camarosa’ or ‘Chandler’ resulted in the absence of product upon PCR amplification in those lines lacking γ-decalactone in their fruits (Sánchez-Sevilla et al. 2014). Therefore, qFaFAD1 is a dominant marker able to identify lines positive for the presence of the *FaFAD1* gene but does not allow discrimination between dominant homozygous and heterozygous lines. In order to test the capacity of this marker to predict the presence of γ-decalactone in strawberry fruits, we PCR tested for specific amplification of *FaFAD1* in the diverse set of 71 strawberry accessions (Fig. 2c). Among them, 30 samples (42.3%) contained the *FaFAD1* allele while the remaining 41 samples (57.7%) were negative in the PCR analysis.

### Evaluation of FaOMT-SI/NO and qFaFAD1 as diagnostic markers for mesifurane and γ-decalactone content in strawberry fruits

Next, we optimized a FaOMT-SI/NO-qFaFAD1 combined test in which three informative alleles can be present: 248 and 217 bp for *FaOMT* and 140 bp for *FaFAD1*. In this multiplexed PCR, the FaOMT-SI/NO products allowed the discrimination between the absence of the *FaFAD1* allele in a sample and total PCR failure.

**Fig. 3:**
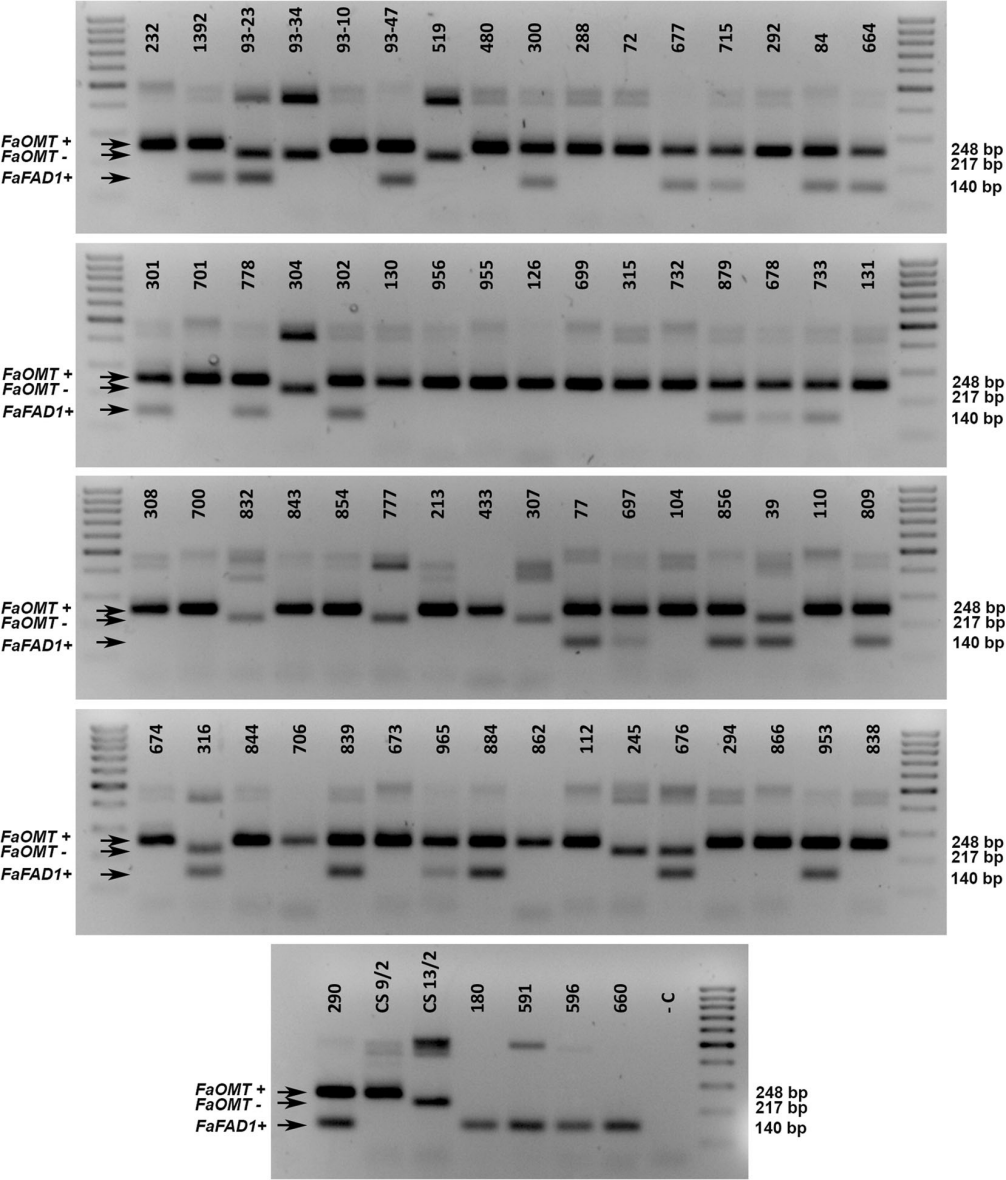
Combined PCR test using *FaOMT* and *FaFAD1* markers for prediction of mesifurane and g-decalactone content in strawberry. Accession numbers of the 71 samples correspond to those described in Table 1. Lane M indicates 100-bp DNA ladder. Lane − C, non-template control

The 71 strawberry accessions were then screened with the combined PCR test, and observed genotypes for *FaOMT* and *FaFAD1* were identical to those generated using single tests for all accessions (Fig. 3). In this combined test, accession homozygous for the 248 bp allele and heterozygous 248/217 were difficult to differentiate. However, for evaluation of test performance, the presence and absence of the 248 bp allele has been used as diagnostic of the presence of the functional *FaOMT* gene. Contingency tables were constructed for both markers using the presence/absence of diagnostic bands summarized in Table 1, and indicators for test performance were calculated (Table 2). The accuracy and ADOR values for the FaOMT-SI/NO marker were 94.03% and 286.1, respectively, indicating a high predictive value for this test. This marker presented maximum values for sensitivity (true positive rate) and NPV, since no false negatives were found (Table 2). Four false positive cases were found corresponding to cultivars ‘Aromas’, ‘Candonga’, ‘Elvira’, and ‘Pedrone’ and resulted in PPV and specificity values of 0.93 and 0.75, respectively (Table 2). These cultivars presented the functional 248 bp allele in either homozygosis or heterozygosis, but none of them contained mesifurane in their fruits. As previously stated, the FaOMT-SI/NO marker cannot be used for the other *Fragaria* species, as predictive bands are not amplified. Among those four wild accessions, three contained mesifurane in their fruits while *F*. *vesca* ‘Reine des vallées’ did not contain detectable amounts (Fig. 3, Table 1).

**Table 2:** Indicators of diagnostic performance for FaOMT-SI/NO and qFaFAD1 marker tests

Marker test	Accuracy (%)	Sensitivity	Specificity	PPV	NPV	ADOR
FaOMT-SI/NO	94.03	1.00	0.75	0.93	1.00	286.1
qFaFAD1	91.55	0.88	0.95	0.93	0.90	129.5

ADOR ranges from 0 to ∞ (the higher the number, the better the diagnostic test)

*PPV* positive predictive value, *NPV* negative predictive value, *ADOR* adjusted diagnostic odds ratio

The γ-decalactone 140 bp *FaFAD1* allele was diagnostic for 65 out of 71 *Fragaria* accessions (Table 1) resulting in a predictive power or accuracy of 91.55%. For this marker, the resulting ADOR value was 129.5 (Table 2). Cultivars ‘Aguedilla’, ‘Parker’, ‘Plarionfre’, and ‘Tioga’ tested negative for the 140 bp allele, and their content of γ-decalactone was below the detection threshold of 6.0. Therefore, we decided to consider these four accessions as non-producers. Among the six accessions with inconsistencies, ‘Galante’ and *F*. *vesca* 596, presented the 140 bp allele but did not contain γ-decalactone in their fruits (0.93 for PPV) while the four remaining accessions (proprietary cultivar 4, ‘Gento’, ‘Oso Grande’, and CS9/2) were negative in the qFaFAD1 test but presented a low, although above threshold content, of γ-decalactone (0.90 for NPV) (Table 2). Other previously described primer pairs spanning the open reading frame of *FaFAD1* (Sánchez-Sevilla et al. 2014) also tested negative in these four accessions, suggesting a deletion of this gene or a radically different sequence in these samples, as previously reported for strawberry accessions without γ-decalactone (Chambers et al. 2014; Sánchez-Sevilla et al. 2014).

### Sequence analysis of *FaOMT* promoter fragment in samples with contradictory results

In order to investigate the inconsistencies observed between the FaOMT-SI/NO marker and mesifurane production, we isolated and analyzed the sequence of the promoter fragment of *FaOMT* amplified by the marker in six accessions including the four false positive samples and two different species: *F*. *virginiana* UC-11 and *F*. *moschata* ‘Capron Royal’. For *F*. × *ananassa* cultivars ‘Aromas’, ‘Candonga’, ‘Elvira’, and ‘Pedrone’, the marker predicted the production of mesifurane (presence of the 248 bp allele) but the fruit did not accumulate detectable amounts. In the accessions *F*. *virginiana* UC-11 and *F*. *moschata* ‘Capron Royal’ that lacked the predictive bands and produced mesifurane, the FaOMT-SI/NO marker produced a unique band of about 500 bp. For these two accessions, we aimed to determine whether cis-acting elements associated with the 30 bp indel in the 248/217 bp alleles were present in this 500 bp allele that could explain mesifurane production in these two *Fragaria* species (Zorrilla-Fontanesi et al. 2012).

Sequences of promoter fragments were compared with the functional and inactive alleles previously published from lines 93-62 and 93-23, respectively (Zorrilla-Fontanesi et al. 2012). As shown in Online Resource 5, high homology in described cis-acting elements was observed in the six sequenced alleles when compared to previously characterized alleles. The promoter sequence from ‘Aromas’, ‘Candonga’, ‘Elvira’, and ‘Pedrone’ was identical to the active 248 bp allele previously characterized from line 93-62 (Online Resource 5). Three specific motifs in this allele have been associated with high *FaOMT* expression and production of mesifurane in fruit (Zorrilla-Fontanesi et al. 2012). The promoter sequences from *F*. *virginiana* and *F*. *moschata* species, whose fruits contain high levels of mesifurane, were more similar in size and sequence to the promoter of *FvOMT* from the *F*. *vesca* reference genome (Shulaev et al. 2011). A 3-bp deletion was observed in these three species in the region where the 30 bp indel is present in *F*. × *ananassa.* None of the three motifs associated with mesifurane production were entirely conserved in these two accessions compared with the active 248 bp allele, as previously shown for the *F*. *vesca* promoter sequence (Online Resource 5; Zorrilla-Fontanesi et al. 2012).

## Discussion

Modern strawberry cultivars are considerably less aromatic than wild species or than the majority of traditional *F*. × *ananassa* varieties (Ulrich et al. 2007; Ulrich and Olbricht 2013). Breeding efforts for the selection of new fruit cultivars during the last two centuries have been dedicated to improve traits that are key for growers such as yield, fruit size, and firmness, and this has indirectly resulted in a deterioration of volatile diversity (Folta and Klee 2016). The majority of breeding programs worldwide are now including the evaluation of sugar content and sugar/acid ratio in their selection process. However, volatile quantification is expensive and time-consuming, and therefore beyond the resources of most breeding programs.

Because volatile patterns are developmentally regulated and strongly dependent on environmental cues (Klee 2010), volatile phenotyping represents a bottleneck for improvement of strawberry fruit flavor and aroma. The development of efficient molecular markers for accurate prediction of desired aroma compounds would greatly accelerate the selection of new cultivars with improved characteristics. In this study, we have optimized a single PCR test for evaluation of functional alleles in two genes, *FaOMT* and *FaFAD1*, which control the production of mesifurane and γ-decalactone, volatiles that contribute to sweet Sherry and fresh peach-like notes to strawberry fruits, respectively (Zorrilla-Fontanesi et al. 2012; Chambers et al. 2014; Sánchez-Sevilla et al. 2014). Next, we evaluated the prediction accuracy of these markers by comparing the presence of functional alleles with the occurrence of the volatiles in fruits using a set of 71 diverse strawberry accessions. Marker FaOMT-SI/NO amplified one to five bands of different length in the selected accessions, in accordance with their polyploid nature. In strawberry cultivars, the production of mesifurane has been associated with the presence of an allele of 248 bp, while a 217 bp allele, due to a 30-bp deletion in the proximal region of the *FaOMT* promoter, causes an inactive allele (Zorrilla-Fontanesi et al. 2012). In this study, approximately 25% of the accessions did not accumulate significant amounts of mesifurane in their fruits, and frequencies of the 248 and 217 bp *FaOMT* allele were approximately 0.5.

As previously reported for other *F*. *vesca* accessions, one product of about 500 bp was present in the two accessions of the diploid *F*. *vesca* tested in this study (Zorrilla-Fontanesi et al. 2012)*.* One or two bands of similar size to that of *F*. *vesca FvOMT* were detected in the hexaploid *F*. *moschata* and the octoploid *F*. *virginiana.* Sequence analysis of the 500 bp promoter fragments of *F*. *moschata* and *F*. *virginiana* has confirmed that the 30 bp deletion is not present in these species, as expected for fruits containing mesifurane. However, the three cis-acting elements specific to the 248 bp allele and associated with mesifurane production in the 232 × 1392 population were not entirely conserved in these two species. Therefore, currently, we lack an explanation for the high content of mesifurane in fruits of these two species, which could be associated with these slightly different motifs, additional unidentified motifs in these diverse and longer promoters or the presence of other genes. The identification of markers associated with the presence/absence of mesifurane in these species awaits future work beyond the scope of this study. Therefore, the absence of predictive bands of 248 and 217 bp using the FaOMT-SI/NO marker in accessions of *Fragaria vesca*, *F*. *iinumae*, *F*. *moschata*, and *F*. *virginiana* (this study; Zorrilla-Fontanesi et al. 2012) suggests that this test is not useful for the prediction of mesifurane in fruits of these species.

Among the *F*. × *ananassa* cultivars, only four (‘Aromas,’ ‘Candonga’, ‘Elvira,’ and ‘Pedrone’) tested positive for the functional 248 bp *FaOMT* allele but lacked mesifurane in their fruits. Analysis of the promoter sequence from these accessions confirmed that the sequences were identical to that of line 93-62, indicative of functional *FaOMT* alleles in these four inconsistent samples. Sequencing of the open reading frame of *FaOMT* in these four accessions would be necessary to rule out the possibility of a non-functional allele despite a functional promoter fragment. A plausible explanation for this non-matching result is the presence of another inactive gene involved in mesifurane biosynthesis in these cultivars. The QTL associated with *FaOMT* controlled from 42 up to 67.3% of the phenotypic variance in a 3-year study (Zorrilla-Fontanesi et al. 2012). Therefore, additional yet unidentified loci could also contribute to the natural variation in mesifurane content in strawberry. Indeed, the *FaOMT* gene encodes the last enzyme in the biosynthetic pathway, and it is possible that a mutation in another up-stream gene limits mesifurane biosynthesis in cultivars with functional *FaOMT* alleles such as ‘Pedrone.’ Pedigree data of these four accessions is limited, and while ‘Aromas’ (Cal. 87.112-6 × Cal. 88-270-1), ‘Candonga’ (Planasa 92-38 × 86-032), and ‘Pedrone’ (not available) may share Californian lines in their pedigree, ‘Elvira’ (‘Gorella’ × ‘Vola’) represents the European ancestry group. Therefore, there is no clear evidence of sharing common sources of a gene mutation. Another possibility is a non-favorable environment for the production of mesifurane in spite of having a functional *FaOMT* allele. In this context, an ongoing study using ‘Candonga’ grown in commercial conditions in Huelva (Spain) has shown accumulation of mesifurane in fruits of this cultivar (unpublished results). Therefore, part of the false positive rate of this marker can be explained by environmental conditions affecting the accumulation of mesifurane in strawberry cultivars. Therefore, the indicators of diagnostic performance of this marker (Table 2) could have been higher if assayed in controlled conditions.

Approximately 42.3% of the tested strawberry lines were positive for the *FaFAD1* gene, consistent with 45% of the accessions producing levels of γ-decalactone above threshold in their fruits. This lactone is one of the most environmentally affected volatiles in strawberry (Olbricht et al. 2011; Chambers et al. 2014). Therefore, one possible explanation for a low number of lines producing γ-decalactone could be that environmental conditions during the collection of fruits from these lines were not favorable for γ-decalactone production. Environmental effects could explain why ‘Galante’ and *F*. *vesca* 596 did not produce γ-decalactone albeit testing positive for the 140 bp *FaFAD1* allele. The four remaining inconsistent results involved samples testing negative for the *FaFAD1* gene but still presenting low but above threshold content of γ-decalactone, which could be explained by existence of an alternative pathway not yet reported. The accuracy, sensitivity, NPV and ADOR values for this marker were slightly lower than for FaOMT-SI/NO marker. However, the diagnostic performance of both markers here described was comparable to that calculated in controlled conditions for marker Bx215_128, associated with perpetual flowering in octoploid strawberry (Salinas et al. 2017).

## Conclusions

Breakthrough developments in the last few years, such as the availability of strawberry genome sequences (Shulaev et al. 2011; Hirakawa et al. 2014; Jung et al. 2014; Tennessen et al. 2014) and the development of high-throughput genotyping platforms for strawberry (Sánchez-Sevilla et al. 2015; Bassil et al. 2015), will greatly accelerate the identification of causal genes responsible for traits of interest. The main bottleneck for development and validation of candidate genes and markers associated with them will be efficient phenotyping, particularly for metabolic or physiologic traits. Untargeted volatile analysis of fruits is therefore one of such bottlenecks. This panel of 71 strawberry accessions has been already phenotyped for a total of 31 different VOCs and represents a useful tool for the validation of candidate markers predicting the production of different volatiles.

Using this diverse set of accessions, we have shown that the combined *FaOMT/FaFAD1* test can predict the production of mesifurane and γ-decalactone in strawberry with high accuracy 94.03 and 91.55%, respectively. When applied for the selection of cultivars with these VOCs in their fruits, the associated error rate compared to selecting by the phenotype would involve only false positives for mesifurane screening, while for γ-decalactone, 3 and 6% would be false positives and false negatives, respectively. However, all false negative samples displayed low content of γ-decalactone, and thus, qFaFAD1 marker is highly efficient for the prediction of strawberry cultivars with high content of γ-decalactone. Therefore, implementation of this DNA test in strawberry breeding programs could facilitate efficient parental selection as well as development of new strawberry cultivars with superior flavor. While agarose-based markers are easy to implement in most laboratories, high-throughput marker systems are more efficient for seedling selection during breeding programs, and thus, the development of multiplexed sets of markers for different traits based on Kompetitive Allele Specific PCR (KASP) or high-resolution melting (HRM) would be a step forward in this regard.

## Electronic supplementary material

Online Resource 1 (PDF 199 kb).

Online Resource 2 (PDF 62 kb).

Online Resource 3 (PDF 95 kb).

Online Resource 4 (XLSX 62 kb).

Online Resource 5 (PDF 109 kb).
