# Supplementary material for: Validation of a PCR test to predict the presence of flavor volatiles mesifurane and γ-decalactone in fruits of cultivated strawberry (Fragaria × ananassa)
Source: Mol Breed. 2017 Oct 2;37(10):131. doi: 10.1007/s11032-017-0732-7 (PMC5624981; doi:10.1007/s11032-017-0732-7)
Supplement: Supplementary file 1 — (PDF 199 kb). [file 11032_2017_732_MOESM1_ESM.pdf]

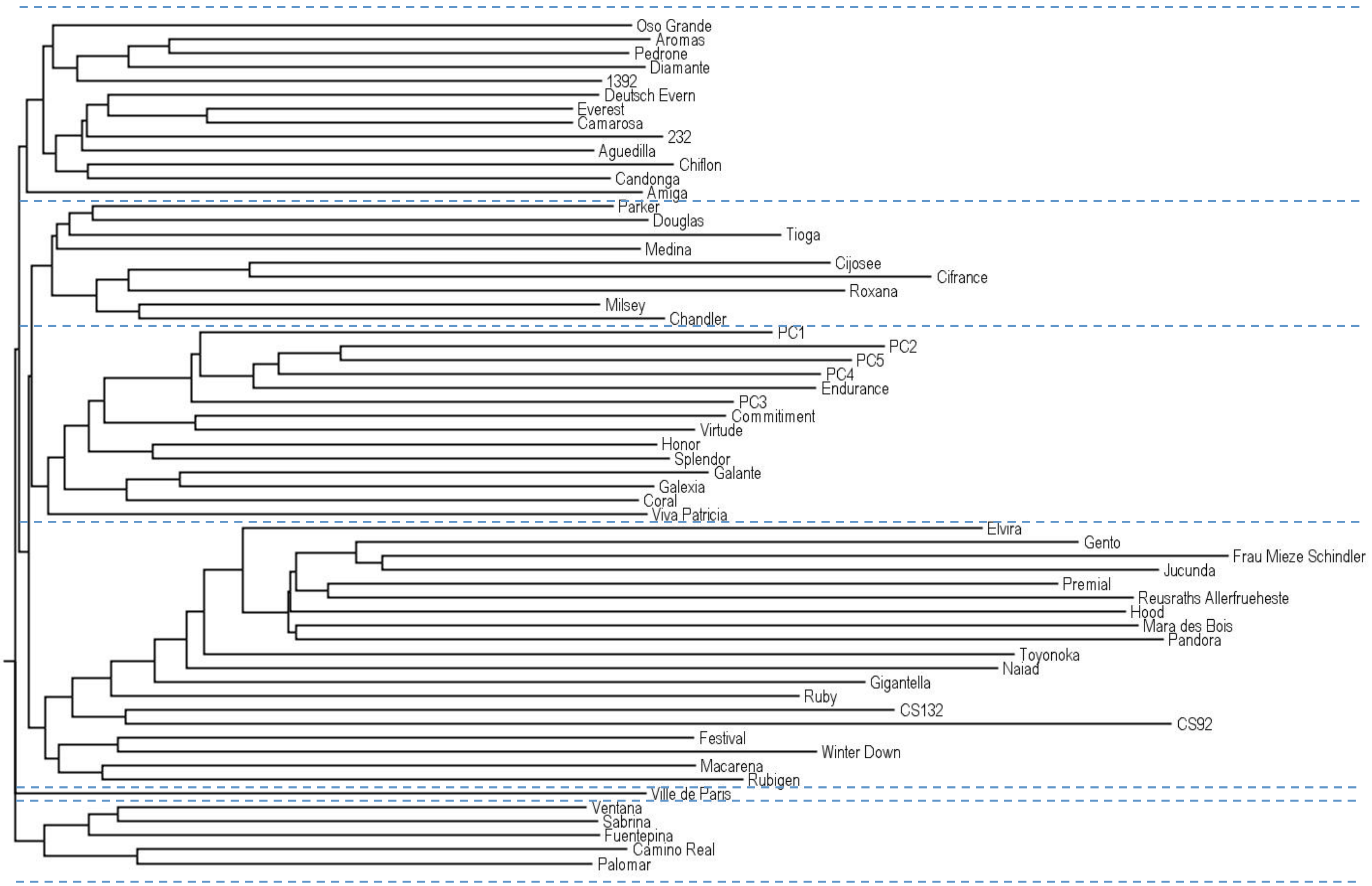

Online Resource 1 Neighbor-Joining clustering of 61 strawberry accessions (out of the 71 used in this manuscript) based on 21,971 SNPs markers. Accessions were selected based in an ongoing study (unpublished results) using a total of 176 accessions genotyped using the Affymetrix IStraw90 Axiom array (Bassil and Davis et al. 2015). The 61 selected accessions were also distributed in different clusters in the complete analysis.
