# Supplementary material for: Validation of a PCR test to predict the presence of flavor volatiles mesifurane and γ-decalactone in fruits of cultivated strawberry (Fragaria × ananassa)
Source: Mol Breed. 2017 Oct 2;37(10):131. doi: 10.1007/s11032-017-0732-7 (PMC5624981; doi:10.1007/s11032-017-0732-7)
Supplement: Supplementary file 2 — (PDF 62 kb). [file 11032_2017_732_MOESM2_ESM.pdf]

Online Resource 2 Primer sequences for markers FaOMT-SI/NO and qFaFAD1, range of alleles and melting temperature (Tm)

Gene	Primer	Sequence 5´-3´	length (bp)	Product (bp)	Tm
FaOMT	FaOMT-SI/NO-F	CGATCATTTCGAAAAGGACTAGT	23	482-217	57.0 ºC
	FaOMT-SI/NO-R	AAGCAGGGTTAGTTGTGGAGA	21		58.8 ºC
FaFAD1	qFaFAD1-F	TCTGTACTCTACCGCCTTGC	20	140	60.0 ºC
	qFaFAD1-R	TCGTAGTGTGGCAGTGAAGG	20		60.0 ºC
