# Supplementary material for: Validation of a PCR test to predict the presence of flavor volatiles mesifurane and γ-decalactone in fruits of cultivated strawberry (Fragaria × ananassa)
Source: Mol Breed. 2017 Oct 2;37(10):131. doi: 10.1007/s11032-017-0732-7 (PMC5624981; doi:10.1007/s11032-017-0732-7)
Supplement: Supplementary file 3 — (PDF 95 kb). [file 11032_2017_732_MOESM3_ESM.pdf]

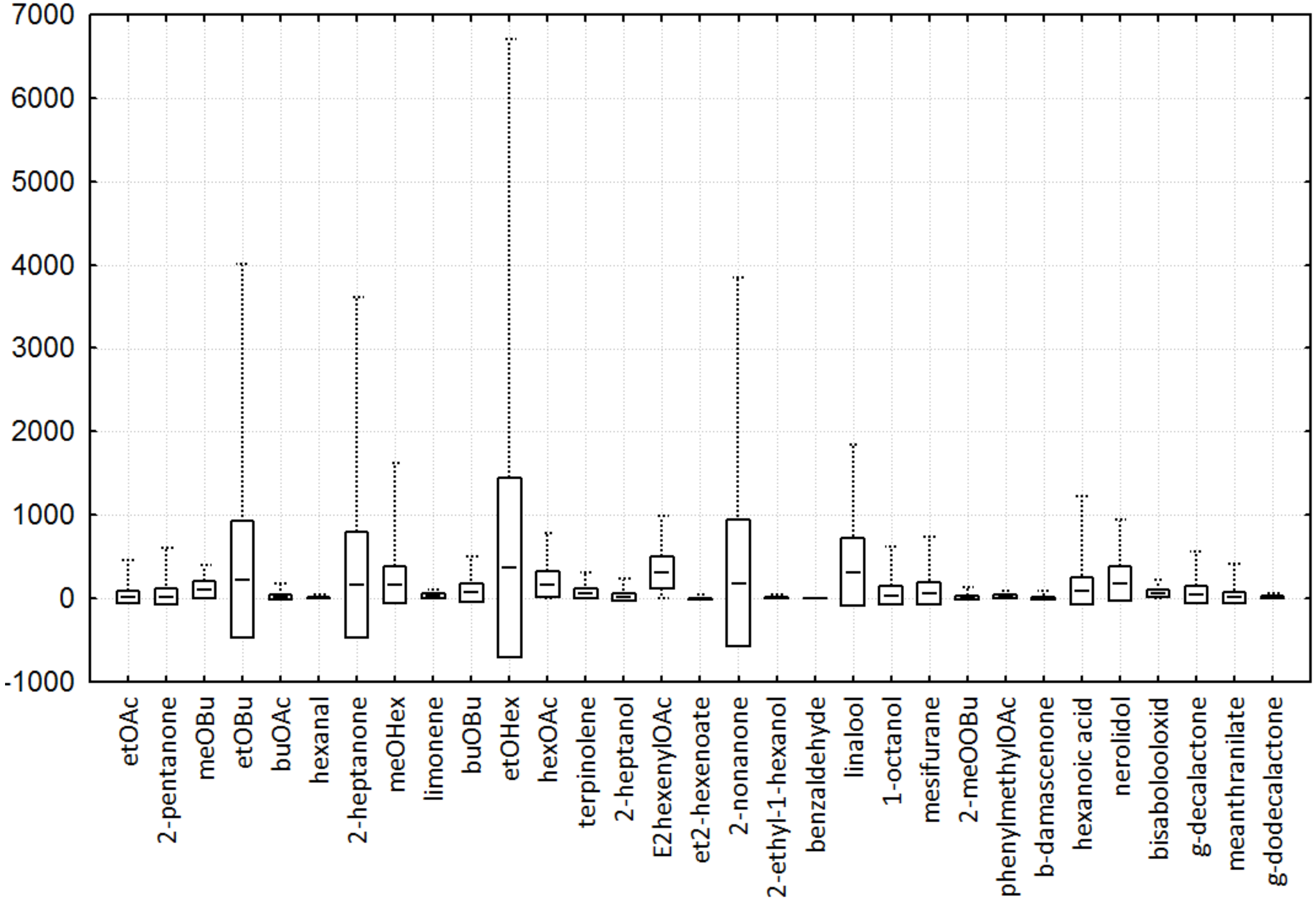

Online Resource 3 Box-plots of 31 VOCs from 60 genotypes. X-axis: 31 VOCs in in the order of retention indices. Box: mean value +/- standard deviation. Whisker: min-max
