# Supplementary material for: Validation of a PCR test to predict the presence of flavor volatiles mesifurane and γ-decalactone in fruits of cultivated strawberry (Fragaria × ananassa)
Source: Mol Breed. 2017 Oct 2;37(10):131. doi: 10.1007/s11032-017-0732-7 (PMC5624981; doi:10.1007/s11032-017-0732-7)
Supplement: Supplementary file 5 — (PDF 109 kb). [file 11032_2017_732_MOESM5_ESM.pdf]

```
                1        10        20        30        40        50        60        70        80        90        100       110       120       130
                |        |         |         |         |         |         |         |         |         |         |         |         |         |
                   WUN/MOTIF
        93-62   CGATCATTTCGAAAAGGACTAG-TGATTGTTTGTGTACTCA-----------------------------------------------------------------------------------------
     'Aromas'   CGATCATTTCGAAAAGGACTAG-TGATTGTTTGTGTACTCA-----------------------------------------------------------------------------------------
   'Candonga'   CGATCATTTCGAAAAGGACTAG-TGATTGTTTGTGTACTCA-----------------------------------------------------------------------------------------
     'Elvira'   CGATCATTTCGAAAAGGACTAG-TGATTGTTTGTGTACTCA-----------------------------------------------------------------------------------------
    'Pedrone'   CGATCATTTCGAAAAGGACTAG-TGATTGTTTGTGTACTCA-----------------------------------------------------------------------------------------
        93-23   TGATCATTACGAAAAGGACTAG-TGATTGTTTGTGTACTC------------------------------------------------------------------------------------------
F. virginiana   CGATCATTTCGAAAAGGACTAGTTAATTGTTTGTGTTCTCAATCTATTATAAGAACCTTTATTATGCGATAATTAATCGTTACTATCTACAAAATTAAATTGGTTGTCAGCCTGCCTTCGTGCCGAAGTT
  F. moschata   CGATCATTTCGAAAAGGACTAG-TAATTGTTTGTGTTCTCAATCT--------AACCTTTATTATGCGATAATTAATCGTTACTATCTACAAAATTAAATTGGTTGTCAGCCTACCTTCGTGCCGAAGTT
 FvOMT g12447   TGATCATTACGAAAACGACTAGTTAATTGTTTGTGTTCTCAATCTATTATAAGAACCTTTATTATGCGATAATTAATCGTTACTATCTACAAAATTAAATTGGTTGTCAGCCTGCCTTCGTGCCGAAGTT
                 ******* ****** ****** * *********** ***

        93-62   ----------------------------------------------------------------------------------------------------------------------------------
     'Aromas'   ----------------------------------------------------------------------------------------------------------------------------------
   'Candonga'   ----------------------------------------------------------------------------------------------------------------------------------
     'Elvira'   ----------------------------------------------------------------------------------------------------------------------------------
    'Pedrone'   ----------------------------------------------------------------------------------------------------------------------------------
        93-23   ----------------------------------------------------------------------------------------------------------------------------------
F. virginiana   ACGGTAAAAAACACGCATCATCTCAAAAGTTTTGTAGAACAGTTACATATTTGGGA------------------TTCACAT----GAGTTGATACAATACTAATTCTACTAAAAATAATTCTCATGTCAT
  F. moschata   ACGGTAAAAAACACGCATCATCTCAAAAGTTTTGTAGAACAGTTACATATTTGGGATCAGTTACATCAATAGTGTTCACATTCAAGAGTTGATACAATACTAATTCTACTAAAAATAAATCTCGTGTCAT
 FvOMT g12447   ACCGTAAAAAACACGCATCATCTCAAAAGTTTTGTAGAACAGTTACATATTTGGGA------------------TTCACATTCAAGAGTTGATACAATACTAATTCTACTAAAAATAATTCTCATGTCAT

                                     GARE-motif   EBOX/RRE           MYBL                                ABRE/ACGT-box                AUXRR-core
        93-62   -----------------------AAACAGAAATGTTCAAATGTACAAGCAAGCCAGTTCCTAGCTAAGTTTAATATAGGATTAAGTAAACTTCACGTATTTGGCAAGTAGGTTCAGGATCATGGACCTA
     'Aromas'   -----------------------AAACAGAAATGTTCAAATGTACAAGCAAGCCAGTTCCTAGCTAAGTTTAATATAGGATTAAGTAAACTTCACGTATTTGGCAAGTAGGTTCAGGATCATGGACCTA
   'Candonga'   -----------------------AAACAGAAATGTTCAAATGTACAAGCAAGCCAGTTCCTAGCTAAGTTTAATATAGGATTAAGTAAACTTCACGTATTTGGCAAGTAGGTTCAGGATCATGGACCTA
     'Elvira'   -----------------------AAACAGAAATGTTCAAATGTACAAGCAAGCCAGTTCCTAGCTAAGTTTAATATAGGATTAAGTAAACTTCACGTATTTGGCAAGTAGGTTCAGGATCATGGACCTA
    'Pedrone'   -----------------------AAACAGAAATGTTCAAATGTACAAGCAAGCCAGTTCCTAGCTAAGTTTAATATAGGATTAAGTAAACTTCACGTATTTGGCAAGTAGGTTCAGGATCATGGACCTA
        93-23   -----------------------AAACAGAAAAGTTTA------------------------------CTTAATATAGGATTAAGTAAACTTCAAGTATTTGGCAAGTAGGGTCAGGATCATGGACCTA
F. virginiana   TAAATTTACAT----CGATGATTAAACAGAATTTTTTAGATGAACAAGGCAGGCAGTTCC---ATAAGTTTAATATAGGATTAAGTAAACTTCAAGTTTTTGGCAAGTAGGGTCAGGATCATGGACCTA
  F. moschata   TAAATTTACATCATACGATGATTAAGCAGAATTTTTTAGATGAACAAGGCTGGCAGTTCC---ATAAGTTTAATACAGGATTAAGTAAACTTCAAGTTTTTGGCAAGTAGGGTCAGGATCATGGACCTA
 FvOMT g12447   TAAATTTACATCATACGATGATTAAACAGAATTTTTTAGATGAACAAGGCAGGCAGTTCC---ATAAGTTTAATATAGGATTAAGTAAACTTCAAGTTTTTGGCAAGTAGGGTCAGGATCATGGACCTA
                                       ** *****   ** *                               ****** ****************** ** ************* *****************

                                                                              TATA-box
        93-62   ATGCTCGACACTTTTGGGTTCCTGCGAGCGCATCAAAGATGGT------------AAGACCACCATATATGTAACCCCCACTCACTATTATCATCTCCACAACTAACCCTGCTT  248
     'Aromas'   ATGCTCGACACTTTTGGGTTCCTGCGAGCGCATCAAAGATGGT------------AAGACCACCATATATGTAACCCCCACTCACTATTATCATCTCCACAACTAACCCTGCTT  248
   'Candonga'   ATGCTCGACACTTTTGGGTTCCTGCGAGCGCATCAAAGATGGT------------AAGACCACCATATATGTAACCCCCACTCACTATTATCATCTCCACAACTAACCCTGCTT  248
     'Elvira'   ATGCTCGACACTTTTGGGTTCCTGCGAGCGCATCAAAGATGGT------------AAGACCACCATATATGTAACCCCCACTCACTATTATCATCTCCACAACTAACCCTGCTT  248
    'Pedrone'   ATGCTCGACACTTTTGGGTTCCTGCGAGCGCATCAAAGATGGT------------AAGACCACCATATATGTAACCCCCACTCACTATTATCATCTCCACAACTAACCCTGCTT  248
        93-23   ATGCTCGACACTTTTGGGTGCCTGCGAGCGCATCAAAGATGGT------------AAGACCACCATATATGTAACCCCCACTCACTATTATCATCTCCACAACTAACCCTGCTT  217
F. virginiana   ATGCTCGACACTTTTGGGTGCCTGGGAGCACATCAAAGATGGTGGTTGGTGAGCCAAGACCACCATATATGTAACCCCCACTCACTCTTATCATCTCCACAACTAACCCTGCTT  474
  F. moschata   ATGCTCGACACTTTTGGGTGCCTGGGAGCACATCAAAGATGGTGGTTGGTGAGCCAAGACCACCATATATGTAACCCCCACTCACTATTATCATCTCCACAACTAACCCTGCTT  491
 FvOMT g12447   ATGCTCGACACTTTTGGGTGCCTGGGAGCACATCAAAGATGGTGGTTGGTGAGCCAAGACCACCATATATGTAACCCCCACTCACTCTTATCATCTCCACAACTAACCCGGCCT  482
                ******************* **** **** *************            ******************************* ********************** ** *
```

Online Resource 5. Sequence alignment of promoter fragments of *FaOMT* in comparison to the *F. vesca* H4 reference sequence. Promoter sequences of active and inactive alleles from lines 93-62 and 93-23 were obtained from NCBI, accession numbers JQ322654 and JQ322651, respectively. Common cis-acting motifs described in Zorrilla-Fontanesi et al. 2012 are shown in blue. Motifs specific to the active 248bp allele are highlighted in red.
